# Metabolomic applied to omalizumab effect in severe asthmatics – a preliminary result

**DOI:** 10.1186/2045-7022-3-S1-P26

**Published:** 2013-05-03

**Authors:** Cláudia Chaves Loureiro, M Caldeira, Sílvia Rocha, Ana Todo-Bom, Mario Loureiro

**Affiliations:** 1 Hospitais da Universidade de Coimbra, Serviço de Pneumologia, Portugal; 2 Universidade de Aveiro, Departamento de Quimica, Portugal; 3 Hospitais da Universidade de Coimbra, Serviço de Imunoalergologia, Portugal

## Background

Omalizumab is a recombinant monoclonal anti-immunoglobulin (Ig)E antibody (Ab), approved for treatment of severe asthma. The mechanisms by which is effective in asthma control are not yet fully understood but is known that it has an anti-inflammatory effect conducting to important clinical benefits. Urine sample collection is the least invasive form for biofluid sampling. The analysis of metabolomic urine profile in asthmatic patients undergoing omalizumab may add important data to the current knowledge.

## Method

Case report: We present the case of a 48 year old woman, with severe persistent allergic asthma, despite level 4 (GINA) medical treatment, who initiated omalizumab in order to control her nocturnal symptoms and her frequent unscheduled medical visits. Before treatment and at 12 weeks: clinical evaluation with ACT was registered; lung function, FeNO, IgE and eosinophils were measured. Two-dimensional gas chromatography (GC ´GC-ToFMS) combined with headspace solid phase microextraction (HS-SPME) was applied to the untargeted study of the volatile metabolomic urine profile.

## Results

The patient showed a good clinical response: ACT improved from 16 to 22, with nocturnal and effort symptoms control, without any unscheduled medical visit, showing a stable lung function, despite an imprudent auto stepped-down inhaler treatment. Regarding metabolomic urine profile, the present work was focused on aldehydes and alkanes (metabolites possibly linked to oxidative stress and/or inflammation processes). Previously to treatment, the urine profile was mainly characterized by alkanes; after treatment aldehydes had a major importance in the characterization of urine composition.

## Conclusions

Beeing alkanes end-compounds in the sequence of oxidation reactions, it can indicate that oxidative state is at a higher extent before treatment, when compared to urine profile after treatment. In spite of being a case-study, the results suggest that the urinary volatile profiles obtained by HS-SPME/GC ´ GC-ToFMS may be useful for differentiating subjects with different physiological conditions, thus making it worth to further explore its diagnostic potential and follow-up therapy effects.

